# Real-World Evidence for Favourable Quality-of-Life Outcomes in Hungarian Patients with Relapsing-Remitting Multiple Sclerosis Treated for Two Years with Oral Teriflunomide: Results of the Teri-REAL Study

**DOI:** 10.3390/ph15050598

**Published:** 2022-05-13

**Authors:** Krisztina Bencsik, Enikő Dobos, Zita Jobbágy, Adrienne Jóri Birkás, Krisztina Kovács, Mária Sátori, Gyula Lencsés, Gabor Bartok, Erika Losonczi, László Vécsei, on behalf of the Teri-REAL Investigators

**Affiliations:** 1 Department of Neurology, Albert Szent-Györgyi Health Centre, University of Szeged, 6720 Szeged, Hungary; bencsik.krisztina@med.u-szeged.hu; 2 Department of Neurology, Szent Imre University Teaching Hospital, 1115 Budapest, Hungary; drdoboseniko@gmail.com; 3 Department of Neurology, Bács-Kiskun County Teaching Hospital, 6300 Kecskemét, Hungary; jobbagyz@kmk.hu; 4 Department of Neurology, National Institute of Clinical Neurosciences, 1145 Budapest, Hungary; dradricamino@yahoo.com; 5 Department of Neurology, Péterfy Hospital and Jenő Manninger National Institute of Traumatology, 1081 Budapest, Hungary; kovacs.krisztina@otvkft.hu; 6 Department of Neurology, Szent Borbála Hospital, 2800 Tatabánya, Hungary; satori.maria@gmail.com; 7 Department of Sociology, Faculty of Humanities and Social Sciences, University of Szeged, 6720 Szeged, Hungary; lencses@socio.u-szeged.hu; 8 Sanofi-Aventis Zrt., 1045 Budapest, Hungary; gabor.bartok@sanofi.com (G.B.); erika.losonczi@sanofi.com (E.L.)

**Keywords:** teriflunomide, multiple sclerosis, quality-of-life, cognition, fatigue, efficacy, real-world study, disability

## Abstract

Relapsing-remitting multiple sclerosis (RRMS) is a degenerative, inflammatory disease of the central nervous system in which symptoms and disability progression vary significantly among patients. Teri-REAL was a prospective, real-world observational study that examined quality-of-life (QoL) and treatment outcomes in a Hungarian cohort of RRMS patients treated with once-daily oral teriflunomide. QoL was assessed at baseline, 12, and 24 months with the Multiple Sclerosis Quality of Life-54 (MSQoL-54) questionnaire. Other measurements included disease progression (Patient Determined Disease Steps [PDDS]), clinical efficacy (relapses), fatigue (Fatigue Impact Scale [FIS]), depression (Beck Depression Inventory [BDI]), cognition (Brief International Cognitive Assessment in MS [BICAMS]), persistence and safety. 212 patients were enrolled (69.1% female, 50.5% treatment naïve), with 146 (69%) completing the study. Statistically significant improvements in subscales of the MSQoL-54 versus baseline were found at Month 12 and Month 24. Significant improvements were also observed for individual components of the BICAMS score at 24 months, while PDDS, FIS and BDI scores remained stable. The mean annualised relapse rate was 0.08 ± 0.32. There were 93 safety events, most of which were mild to moderate. Improved QoL and cognitive outcomes in teriflunomide-treated patients over 2 years offer a unique perspective to this real-world study.

## 1. Introduction

The past decade has seen a major increase in the number of disease-modifying therapies (DMTs) approved to treat multiple sclerosis (MS), a chronic autoimmune-driven neurodegenerative disease of the central nervous system that affects more than 2.8 million people worldwide [1]. Most of these therapies are indicated for the treatment of relapsing-remitting MS (RRMS), the main disease phenotype that is characterised by episodes of relapse activity followed by full or partial recovery, and a gradual progression of underlying disability [2]. RRMS is a highly heterogeneous condition that affects people differently, not only in terms of the degree of disease activity and the nature and severity of physical affectation, but also in terms of non-physical symptoms such as anxiety, depression, cognitive impairment, mood disorder, irritability, and anger that may severely curtail normal daily functioning and thereby impact on overall quality of life (QoL) [3,4]. There is growing awareness in the medical community that all symptoms, and not just the physical ones, need to be adequately monitored and controlled as part of the patient management process if a patient’s overall wellbeing and QoL is to be preserved [5,6].

Most of the current first-line therapies for MS consist of injectables (subcutaneously- or intramuscularly-administered with pre-filled syringes or pens) and oral DMTs. Teriflunomide is a reversible, selective inhibitor of dihydroorotate dehydrogenase (DHODH), a rate-limiting mitochondrial enzyme required for de novo pyrimidine synthesis by proliferating lymphocytes. Inhibition of this enzyme by teriflunomide is thought to reduce the number of activated T and B lymphocytes that cross the blood-brain barrier and provoke damage within the central nervous system [7,8]. Oral teriflunomide (Aubagio^®^) received marketing approval in Europe in 2013 for the treatment of RRMS [9], providing patients with a more convenient mode of administration and avoiding injection site adverse reactions. Prior to marketing approval, the drug underwent one of the most extensive clinical development programs ever conducted on RRMS patients, with significant clinical efficacy and brain imaging outcomes obtained across phase 2 and 3 clinical trials and their long-term extension studies [7,8,10,11,12,13,14].

In answering questions about the real-world value of a DMT, numerous factors in addition to the benefit-risk characteristics and price of each drug come under consideration. To this end, health economics outcomes data are becoming increasingly important in the public health system and are commonly employed by healthcare authorities and payers in the decision-making process. In line with these requirements, real-world outcomes with teriflunomide have been reported for a number of observational studies conducted mainly in the US and Europe on RRMS patient populations treated with the drug according to local prescribing guidelines [15,16,17,18,19,20,21,22]. Importantly, from a real-world perspective, the patient cohorts enrolled in such studies are not required to conform to the strict inclusion/exclusion criteria specified in randomised clinical trials and therefore represent a broader and more characteristic patient base on which teriflunomide is used.

Teri-REAL was a prospective, real-world observational study carried out from 2016 to 2020 in Hungary, a country with an MS prevalence of approximately 101.8/100,000 persons according to epidemiological data [23]; this extrapolates to around 10,000 Hungarian patients with MS, 69% of whom have RRMS. The objective of Teri-REAL was to generate patient reported outcome (PRO) data to assess QoL, fatigue, depression, disability, and cognitive outcomes in a cohort of around 200 RRMS patients treated with teriflunomide for two years according to the approved European indication. In addition to PRO measures, treatment adherence and persistence, health economics outcomes, and efficacy and safety measures were also assessed both in treatment-naïve patients and in patients previously treated with other DMTs.

## 2. Results

### 2.1. Baseline Characteristics

**Figure 1:**
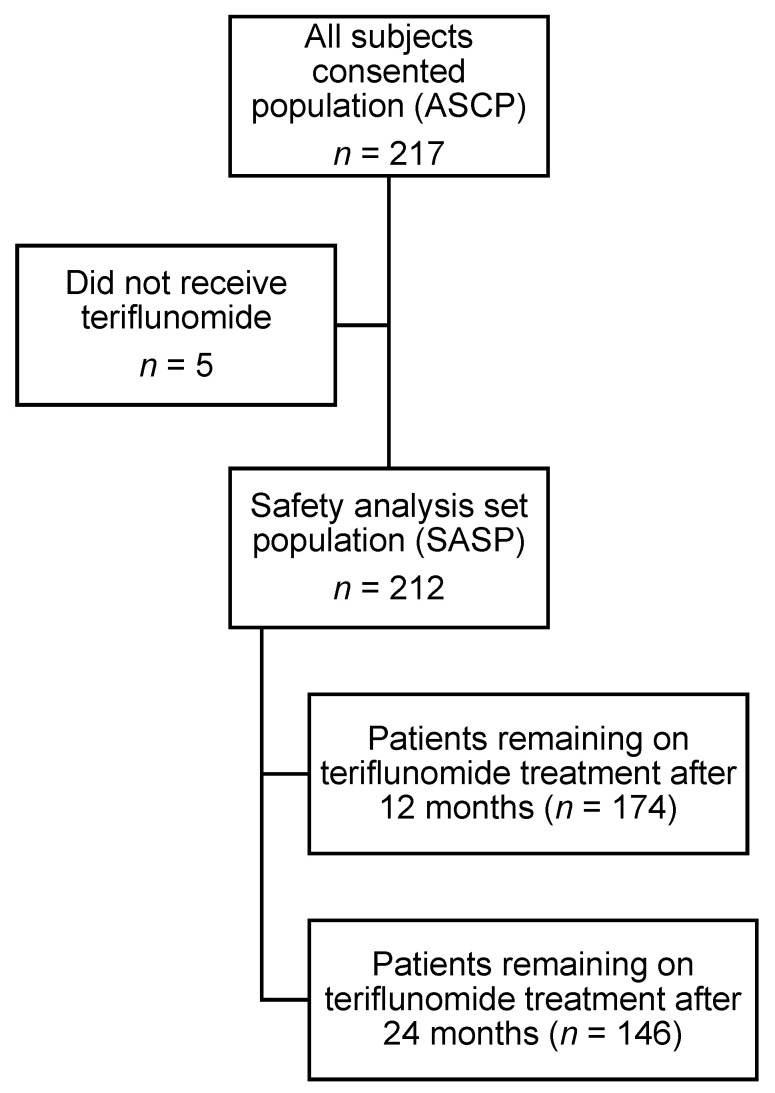
Patient enrolment and outcomes in Teri-REAL.

Teri-Real enrolled 217 patients from 33 clinical sites. As five of the enrolled patients did not proceed to teriflunomide treatment, the Safety Analysis Set Population (SASP) and Full Analysis Set Population (FASP) populations thus consisted of 212 patients (Figure 1).

Baseline patient characteristics (All Subjects Consented Population; ASCP) are presented in Table 1. The mean age (±SD) of patients was 41.8 ± 9.6 years, and 150 of the 217 patients (69.1%) were female. Here, 109 patients (50.2%) were treatment-naïve. Of those who had received treatment with a DMT prior to participating in the study (*n* = 107), more than 80% had been prescribed first-line injectable drugs. One patient had previously been treated with two different DMTs.

Most patients (215 [99.5%]) identified as Caucasian, and more than 70% reported being in full- or part-time employment. At baseline, the mean number of relapses (±SD) over the 2 years prior to study enrolment was 1.04 ± 0.92, and the mean expanded disability status scale (EDSS) score was 1.97 ± 1.40.

### 2.2. Study Discontinuations

In this case, 174 patients (82%) and 146 patients (69%) remained on teriflunomide at 12 and 24 months, respectively, corresponding to a total of 66 discontinuations over the study period from the original 217 patients in the ASCP. Reasons for discontinuations are summarised in Table 2.

Additional analyses showed that there was no significant difference in discontinuation rates between previously treated and treatment-naïve patients (Chi-squared test: *p* = 0.306), nor in relation to the initial EDSS score (analysis of variance (ANOVA): *p* = 0.841).

The two patients who had 4 relapses in the two years prior to enrolment (Table 1) discontinued the study before the month 12 visit, whereas the six patients who had had three relapses each before enrolment completed the study. Patients with 0, 1, or 2 relapses discontinued at approximately the same rate in the first and second years of the study.

### 2.3. Primary Outcome Measure

**Figure 2:**
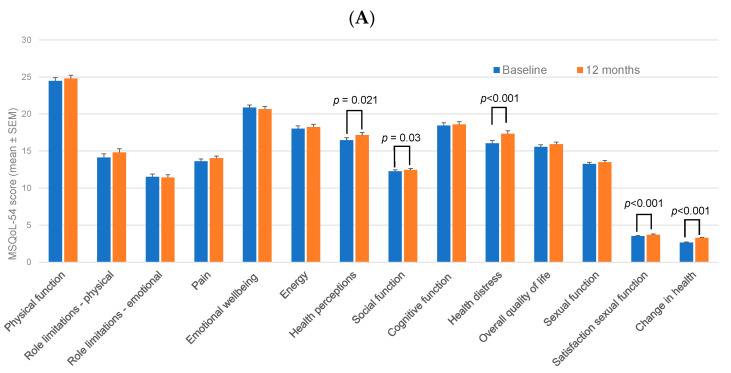

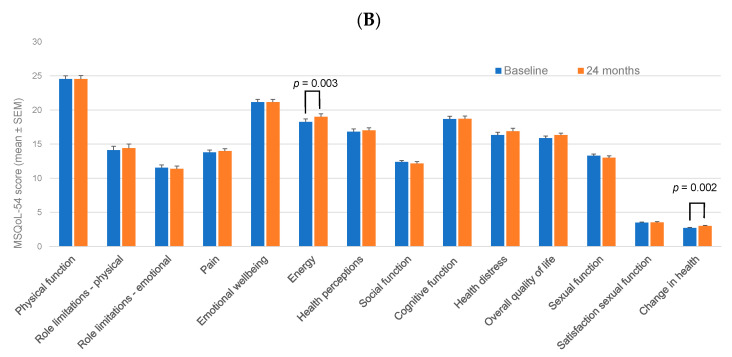
MSQoL-54 scores at Month 12 (**A**) and Month 24 (**B**) compared with baseline. Graphed values show mean ± SEM for each component of the MSQoL-54 test. Statistically significant outcomes from paired sample *t*-test results at 12 months (**A**) and 24 months (**B**) versus baseline are shown. SEM: standard error of the mean.

Results from the subscales of the Multiple Sclerosis Quality of Life-54 (MSQoL-54) questionnaire following 12 and 24 months of teriflunomide treatment are presented in Figure 2. Most of the parameters were stable over the course of the study, with statistically significant improvements (paired samples *t*-tests) at Month 12 versus baseline observed for health perceptions (*p* = 0.021), social function (*p* = 0.03), health distress (*p* < 0.001), satisfaction with sexual function (*p* < 0.001), and change in health (*p* < 0.001) categories (Figure 2A). For the 24-month time-point versus baseline (Figure 2B), significant differences were found for change in health (*p* = 0.002) and energy (*p* = 0.03). Results for the one-way ANOVA (baseline to 2 years) echoed the *t*-test results, with statistically significant improvements seen in MSQoL-54 subscales for social function (*p* = 0.001), health distress (*p* = 0.001) and change in health (*p* < 0.001). Most of the differences seen between the baseline and 12- or 24-month values were small, even when statistically significant. Overall, no statistically significant worsening of any MSQoL-54 subscale parameter was observed, suggesting that QoL in this patient cohort remained stable or improved slightly during the 24-month study period.

### 2.4. Secondary Outcomes

#### 2.4.1. Relapses

In total, 31 relapses were recorded in 28 of the 212 patients (3 patients had 2 relapses) over the 2 years of the study. In this case, 16 of these relapses occurred in 16 patients in the first year of treatment, with 4 of the 16 patients suffering at least 1 relapse in the 2 years prior to the study, and 10 of these patients suffering at least 2 relapses in the 2 years prior to the study. Here, 15 of the 31 relapses occurred in 13 patients in the second year of treatment (2 patients had 2 relapses) with 9 of these 13 patients suffering at least 1 relapse in the 2 years prior to the study, and the remaining 4 patient suffering at least 2 relapses in the 2 years prior to the study. The only patient who had 1 relapse both in year 1 and in year 2, had 1 relapse in the 2 years prior to the study. In this case, 11 of the 16 patients who had relapses in the first 12 months of teriflunomide treatment did not continue to the study’s second year (i.e., no Visit 4 data are available for these patients). Two of the 13 patients who had relapses in the second 12 months of teriflunomide treatment had no data for Visit 4.

The severities of 16 relapses were noted in eCRFs and recorded in the clinical study report. Five of these relapses were classified by the investigator as mild and 11 as being of moderate severity. Patients were hospitalized for 12 of the 16 relapses, and steroid treatment required in 13 of the cases. Recovery was complete for 7 relapses and incomplete for 9 relapses. Taken together, the annualised relapse rate (ARR) (mean ± SD) decreased from 0.73 ± 0.76 in the year prior to baseline measurements, to 0.06 ± 0.24 and 0.08 ± 0.32 at 12 and 24 months, respectively, following teriflunomide treatment initiation (*p* < 0.001 at Months 12 and 24 versus the pre-baseline ARR (repeated ANOVA for post hoc pairwise comparisons).

#### 2.4.2. Patient Determined Disease Steps (PDDS)

**Figure 3:**
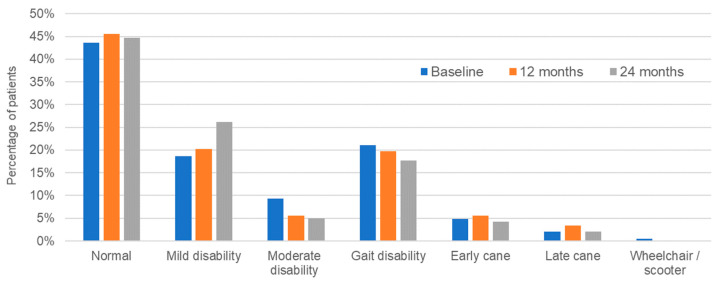
Percentages of patients at each PDDS level from baseline to 2 years.

Compared to the EDSS, the PDDS tool provides a quick, simple to use though nonetheless reliable means for neurologists to measure long-term disease progression in MS based primarily on the assessment of ambulation [24,25]. Results from the PDDS were stable, with no clinically important decrease in score (i.e., disability improvement) observed over the course of the study. That is, the mean PDDS score at baseline was 1.33 [95% CI: 1.13–1.54], compared to 1.3 [95% CI: 1.08–1.52] at the 12-month visit and 1.17 [95% CI: 0.94–1.40] at 24 months (*p* = 0.508; repeated measures ANOVA). Concerning changes in the distribution of disability impairment during the study, 89 patients out of 204 (43.6%) had a normal PDDS score at baseline compared to 63 out of 141 (44.7%) at 24 months. Figure 3 shows the numbers of patients in the different PDDS disability categories expressed as a percentage of the population at each timepoint. Percentages of patients with no disability (normal) or mild disability increased slightly with time on treatment, whereas those with moderate or more severe disability remained constant or tended to decrease during the study. No patients transitioned to ‘wheelchair’. As the results only contemplate patients who remained in the study, it could be expected that the distribution of patients would skew towards those whose disease was under control and therefore had little or no disability. Declining numbers of patients with more serious disability could be due to treatment discontinuation owing to a perceived lack of drug efficacy as in the case of disability worsening.

Values shown are expressed as a percentage of the number of patients at each timepoint.

#### 2.4.3. Fatigue Impact Scale (FIS)

The total FIS score, and the cognitive and psychosocial components of FIS were stable over the course of the study, with none of these measures found to be statistically different from baseline values. The physical component of FIS did show a statistically significant improvement (*p* = 0.008, one-way ANOVA) after 2 years versus baseline (11.33 versus 13.09).

#### 2.4.4. Beck Depression Inventory (BDI)

The mean BDI score improved slightly during the study in patients who had valid BDI scores at baseline and at subsequent visits. In this way, a decrease from 9.505 at baseline to 8.177 was observed at the 12-month visit (*p* = 0.005), while a decrease from 8.528 at baseline to 7.867 was seen at 24 months, though this change was not significant (*p* = 0.182). Taken together, these findings indicate stability in this measure of depression in patients who remained in the study.

#### 2.4.5. Brief International Cognitive Assessment in MS (BICAMS)

Table 3 shows that statistically significant improvements were observed for the three components (Symbol Digit Modalities Test (SDMT), Brief Visuospatial Memory Test (BVMT), California Verbal Learning Test II (CVLT)) of the BICAMS score (*p* < 0.05; paired *t*-tests) after 2 years. The change from baseline to Month 12 and to Month 24 was also significant for each component as measured by one-way ANOVA (*p* < 0.05).

#### 2.4.6. Health Economics Outcomes

Participant employment status improved slightly over the course of the study, with the percentage of study participants in full- or part-time employment and who remained on treatment increasing marginally from 72% to 78%, although this change was not statistically significant (Table 4; Chi-squared test: *p* = 0.181). In this case, 89% of patients who were employed full time at baseline and who remained in the study were still in full-time employment at the 24-month measurement. Moreover, for patients who completed the study, 24% of those who were unemployed at baseline had entered into full-time work, while 12% were in part-time work at the 24-month visit.

There was little change in the number of medical consultations that participants required over the course of the study compared with the 12 months prior to baseline. However, the need for inpatient care (based on visits made in the previous 12 months (mean ± SD)) decreased from 3.3 ± 5.7 at the baseline reading to 1.3 ± 11.6 at 12 months and 1.6 ± 12.0 at 24 months (*p* = 0.189; repeated measures ANOVA). This outcome, though not significant, was skewed by the very high number of visits made by one patient. When the data from this outlier were removed from the analysis the result was statistically significant (*p* < 0.001). Patient absenteeism (missed work, school, or regular daily activities in the previous 12 months) also decreased. The value at baseline was 12.2 ± 43.4 compared to 8.4 ± 41.7 at 12 months and 2.5 ± 12.5 at 24 months (*p* = 0.0497; repeated measures ANOVA).

#### 2.4.7. Adherence and Persistence

Adherence to teriflunomide was high and consistent at both the 12-month visit (97.2%) and the 24-month visit (97.3%). As described above, treatment persistence was also very good, with 82% and 69% of patients still on teriflunomide at 12 and 24 months, respectively. Of the 212 patients in the SASP, exactly half (*n* = 106) had been previously treated and half were treatment-naïve. After 24 months of the study, 75.5% of the previously treated and 66% of the treatment-naïve patients remained on teriflunomide treatment (*p* = 0.306; Chi-squared test).

#### 2.4.8. Safety

##### Clinical Laboratory Tests

There were no anomalous laboratory test results deemed by the investigators to be clinically significant.

##### Adverse Events

There were 93 safety events reported in 44 patients (20.75%) in Teri-REAL. The most frequently reported events were hypertension (6 patients), alopecia (5 patients), and upper respiratory tract infection (4 patients).

Serious AEs occurred during the course of the study in 8 patients (3.77%; one occurrence each of: abdominal pain, anxiety, bipolar disorder, hypersensitivity, multiple sclerosis relapse, thermal burn, tumefactive multiple sclerosis and uterine leiomyoma, of which 4 were classified as moderate and 4 as severe events). Seven patients were hospitalized, 6 recovered (outcome for the other patient is unknown). Teriflunomide was withdrawn from the patient with the severe skin reaction as this was judged to be drug-related.

In total, 13 AESIs (14% of total number of AEs) occurred in 10 patients during the study (medically significant increases in blood pressure [4 events], upper respiratory tract infection [2 events], one occurrence of each of the following: liver disorder with increases in transaminases and bilirubin, severe skin or allergic reaction, major depressive disorder and suicidal attempt, uterine dilation and curettage, frequent bowel movements, bipolar disorder, pain in extremity, and carpal tunnel syndrome. One patient had a psychotic episode, which was caused by a known bipolar affective disorder, and was classified as a SAE.

## 3. Discussion

The Teri-REAL findings are in line with other observational studies evaluating treatment outcomes in unselected teriflunomide-treated patients in real-world clinical settings [15,16,17,18,19,21]. The fact that many of these studies are country- or region-specific provides healthcare professionals and decision-makers in public office with valuable close-up snapshots of treatment outcomes for broadly based patient profiles coupled with different local prescribing practices and drug reimbursement policies across those jurisdictions. This is the first such study to be carried out on teriflunomide-treated RRMS patients from Hungary.

Compared to other real-world studies on teriflunomide-treated patients, the patient profile of the Teri-REAL cohort at baseline can be described as relatively young and with low disability. In this way, the mean age of patients (41.8 ± 9.6 years) is at the lower end of what has been described in several other analogous real-world studies, where mean baseline ages close to or over 45 years were reported [15,16,17,18,19,21]. Along the same lines, the mean EDSS score at baseline (1.97 ± 1.40) was low in Teri-REAL compared to that generally seen in other studies, which in some cases registered baseline EDSS values close to or exceeding 3 [15,18]. From this starting point, the probability of detecting wholesale changes in the QoL primary endpoint or other measures over a 24-month period in our cohort is likely to be reduced. Nevertheless, the baseline demographics of the patient cohort cannot be controlled for a priori in a study of this type, as patients who fulfilled the local prescribing criteria were eligible for enrolment. Although our aim was to evaluate outcomes in an unselected, real-world cohort, further patient-relevant data could be obtained by performing sub-analyses of data from patients grouped according to defined baseline criteria (for example, EDSS > 3.0, prior treatment, etc.). Such analyses are however beyond the scope of the present report.

A key factor to be considered in real-world studies such as that described here is the percentage of patients who discontinue treatment prior to the end of the study. Even if a battery of study tests is carried out at the time of patient withdrawal and data allocated to the nearest planned study visit, discontinuations will tend to cause a bias by eliminating patients who do not respond well to a drug, thus impacting on subsequent analyses. Moreover, obtaining such data at withdrawal is often not feasible for all discontinuing patients in an observational study, meaning that key information about drug efficacy, tolerance, side effects, and treatment compliance among other parameters is not compiled. This characteristic of observational studies should be kept in mind when considering the outcomes of such studies.

We showed here that 69% of Teri-REAL subjects remained on teriflunomide treatment at the end of the study (24 months). This value fits with previously reported real-world teriflunomide studies, where discontinuation rates after 2 years were 29.2% in Italy [18], 34.6% in France [21], 21.5% in Germany [17], 41% in Nordic countries [16], and 24.8% in Greece [19]. Apart from 9 of the 66 discontinuations being due to AEs, detailed underlying reasons for the remaining discontinuations (patient/investigator decision, lost to follow-up, etc) were not compiled in the study database.

The primary outcome measure in Teri-REAL was improved quality of life as determined by the MSQoL-54 test. Small improvements were seen in some subsections of the test at 12 and 24 months, but overall, most parameters remained stable, with no statistically significant worsening seen (Figure 2). These findings are consistent with QoL outcomes from other real-world studies with teriflunomide-treated patients, which showed stable QoL or small improvements in outcomes [15,16,17,19,21]. Whether the observed improvements reported here translate into clinically important differences is not clear; however, the improvement in some parameters and overall absence of worsening shows that the health-related QoL (HRQoL) of patients who remained in the study was maintained.

Teriflunomide treatment provided solid efficacy outcomes in our cohort, with only 13 of the original 212 patients having a relapse, and over 80% of these relapses occurring in the first year of treatment. A decrease in the ARR from 0.73 ± 0.76 in the year prior to baseline measurements to a very low 0.08 ± 0.32 in the second year of teriflunomide treatment confirms or even surpasses results from other teriflunomide-treated cohorts [15,16,17,18,19,21,22]. We found that the efficacy measure for patient-reported disability, as evaluated by PDDS, remained stable over the course of the study. This result could be anticipated given that patients had low disability levels upon entry to the study, and relapse activity was well controlled in those who remained on treatment. Similar stable PDDS outcomes in teriflunomide treated patients have been reported [15,19].

Fatigue and depression are patient symptoms that are known to contribute to disease burden and decreased QoL in MS patients [23,26]. We measured stable total FIS scores and cognitive and psychosocial components, along with a small but statistically significant improvement in the physical component. To place this in context, Rendas-Baum et al., (2010) [27] calculated that the minimum improvement in FIS total score to achieve a clinically meaningful change was 10–20 points. Likewise, depression scores in our study were stable or underwent small yet significant improvements, but these were well short of the almost 20% improvement from baseline BDI scores needed to be considered clinically meaningful [28].

Our findings concerning fatigue and depression measures are again consistent with previous reports for teriflunomide-treated patients [17,21]. Importantly, the stable outcomes described here for these parameters suggest an absence of their deterioration over the 24 months of the study period. This was likely reflected in the overall stable HRQoL of our patient cohort.

The validated Hungarian version of the BICAMS test used in the Teri-REAL study has been described as a “reliable method for the evaluation” of cognitive function in MS patients [29], while Filser et al., (2018) [30] described BICAMS as a reliable monitor of cognitive performance over time. To this end, exceptionally good cognitive test outcomes were obtained in those patients who remained on treatment for the 24 months of the Teri-REAL study, with small though significant improvements determined for all three cognitive tests in the BICAMS battery. Sandi et al., (2015) [29] also showed that performance in the BICAMS test was correlated with 7 of the MSQoL subscales, which may explain in part the stable HRQoL outcomes we report for Teri-REAL.

Cognitive deficits are strongly related to changes in work performance and status [31]. With the study by Filser et al., (2018) [30] noting that the SDMT has good predictive value for working ability, the significant SDMT findings reported here may reflect the slight (though not significant) increase in the number of teriflunomide patients in full- and part-time work at study end. Other health economic outcomes were also positively impacted during the study, with decreased incidences of inpatient care and absenteeism. Furthermore, no new safety signals were seen in Teri-REAL, with the most frequent AEs being alopecia, hypertension, and upper respiratory tract infection, as reported in the clinical trial program and other post-marketing studies.

In conclusion, the results from Teri-REAL are in line with previously published RWE findings and underpin the well-characterized benefit-risk profile of teriflunomide. Improved HRQoL, fatigue, depression, and cognitive outcomes in these patients over 2 years offer a unique perspective to this real-world study.

## 4. Materials and Methods

### 4.1. Study Design

This was a 2-year, prospective, observational, real-world study of teriflunomide-treated RRMS patients in Hungary. Both treatment-naïve and pre-treated patients were eligible for the study, which began enrolling participants on 28 June 2016 (first patient in) and completed on 16 July 2020 (last patient out). It was planned for around 200 patients to be recruited from 33 study sites in Hungary, all of which were staffed by physicians experienced in the diagnosis, management, and treatment of relapsing MS patients with disease-modifying drugs, including teriflunomide, as part of their daily clinical practice.

All outcome measures obtained from patients were assessed at baseline and then at 12 (±1) months and 24 (±1) months. Clinical screening data were collected by the treating neurologist on electronic case report forms (eCRFs). PRO measures were collected from patients who completed hard copy questionnaires, the results of which were then entered into the study database.

This study was conducted in compliance with all international guidelines, national laws, and regulations, including local data protection rules. In addition, the study was conducted in accordance with the ethical principles of the Declaration of Helsinki (1964 and subsequent iterations). Ethics approval for this real-world, observational, non-interventional study was provided by the Hungarian National Institute of Pharmacy and Nutrition (OGYEI/12590-4/2016).

### 4.2. Patients

#### 4.2.1. Inclusion/Exclusion Criteria

Enrolled patients were required to be at least 18 years of age and to have a diagnosis of RRMS as confirmed by the treating neurologist at the participating study site. Inclusion to the study was also dependent on patients being eligible for treatment with teriflunomide (14 mg) according to the approved European Summary of Patient Characteristics [9]. All patients (or their carers) provided written informed consent to participate in the study.

#### 4.2.2. Discontinuations

In the event of a patient choosing to discontinue their involvement in the study, the reason(s) for this discontinuation, if available, was recorded on eCRFs. Patients who discontinued the study did not undergo a final evaluation at that time unless the discontinuation coincided with one of the planned study visits.

### 4.3. Endpoints

The primary endpoint was quality of life as measured with the MSQoL-54 questionnaire (Table 5; [32]) adapted for Hungarian native speakers [33]. This questionnaire consists of 54 questions related to physical health (actual physical function, role limitations due to physical problems, pain, health perception, energy/fatigue, sexual function, social function, health distress) and mental health (health distress, overall quality of life, emotional well-being, role limitations due to emotional problems, cognitive function). Scores for each scale range from 0 to 100, with a higher score indicating improved quality of life.

Secondary endpoints assessed in Teri-REAL (Table 5) consisted of efficacy measures for teriflunomide (ARR and disease progression (measured using PDDS [24,25]), as well as fatigue (FIS [34,35]), depression (BDI [37]), and cognition (BICAMS [39]) validated for Hungarian native speakers [29].

Health economic outcomes related to teriflunomide were assessed from a number of perspectives. Changes in employment status (full-time, part-time, not employed) and absenteeism (the number of days participants missed work, school, or regular daily activities) versus baseline values were evaluated at the 12- and 24-month timepoints with the Health-Related Productivity Questionnaire (HRPQ) [40]. Concomitant medication use, the number of medical consultations over the study period, and the number of days in which participants required impatient hospital care were also assessed. Adherence to teriflunomide was calculated as a self-reported measure according to Osterberg and Blaschke (2005) [41], with patients considered to be adherent if the number of teriflunomide tablets taken over a specified period of time was >80% of the prescribed number that should be taken during that time.

Safety outcomes, including laboratory tests and adverse events (AEs), were also assessed.

### 4.4. Statistical Methods

#### 4.4.1. Populations

The All Subjects Consented Population (ASCP) consisted of all patients who consented to participate in the trial; those who did not start treatment were omitted from the SASP and the FASP.

#### 4.4.2. Methodology

Demographics and baseline characteristics were summarized in a descriptive manner using the mean and SD where appropriate, and percent for categorical data.

For the MSQoL-54, the primary analysis was the comparison of quality of life from baseline to 12 months and baseline to 24 months. Data were analysed descriptively using the mean, median and SD. A paired samples *t*-test was applied to compare baseline and data collected at the 12- and 24-month visits. One-way ANOVA with repeated measures were used to compare the changes during the study. Each of the 14 subsections of the MSQoL-54 was analysed independently. The same statistical analyses were applied to the PDDS, FIS, BDI and BICAMS questionnaire data.

The HRPQ was analysed by Chi-squared test, and adherence to teriflunomide treatment was determined by the number and frequency of adherent patients at each visit. Persistence was calculated as the percentage of patients who remained on treatment at the 12- and 24-month visits.

Adverse events were analysed in a descriptive manner, with the frequency and percentage of patients experiencing AEs, serious AE (SAEs), and adverse events of special interest (AESIs) during the study calculated for each study visit, and summarized by intensity, possible relationship to teriflunomide, outcome and action taken.

## 5. Conclusions

The Teri-REAL findings suggest favourable real-world patient-reported efficacy and safety outcomes with teriflunomide in this Hungarian RRMS population. The study’s results are consistent with PRO outcomes in teriflunomide-treated RRMS patients reported for other observational studies conducted in the US and Europe. The improved QoL and cognitive outcomes in these patients over 2 years offer a unique perspective to this real-world study.

## Figures and Tables

**Table 1:** Baseline characteristics of the Teri-REAL patient cohort (ASCP) ^†^.

Parameter *	Baseline Score	Number
Age, mean (SD), range, years	41.8 (9.6), 18–73	217
Females, *n* (%)	150 (69.1)	217
Race Caucasian, *n* (%)	215 (99.5) ^1^	216
Highest educational level attained, *n* (%) Primary school Secondary school Advanced vocational qualification Graduate	20 (9.2) 103 (47.5) 35 (16.1) 57 (26.3)	217
Employed, *n* (%) ^2^ Full-time Part-time	135 (62.2) 24 (11.1)	217
Time since first MS symptoms were noticed (years)	9.9 (8.2)	216
Time since diagnosis of MS, years	7.3 (7.1)	216
Number of relapses in the past 2 years, mean (SD)	1.04 (0.92)	216
Frequency of relapses in the past 2 years, *n* (%) 0 1 2 3 4	75 (34.6) 68 (31.3) 65 (30.0) 6 (2.8) 2 (0.9)	217
MS treatment naïve at baseline, *n* (%)	109 (50.4)	216
Most common DMT at baseline, *n* (%) Interferon beta 1b Glatiramer acetate Interferon beta-1a (SC) Dimethyl fumarate Interferon beta-1a (IM)	108 (49.8) 28 21 20 20 19	
EDSS score, mean (SD)	1.97 (1.40)	216
PDDS score, mean (SD)	1.33 (1.48)	204

* mean (SD), unless stated; ^†^ All subjects consented population (ASCP); ^1^ One patient did not respond to this question; ^2^ 56 patients were not employed, while 2 patients did not respond to this question. DMT, disease modifying treatment; EDSS, Expanded Disability Status Scale; IM: intramuscular; MSIS, Multiple Sclerosis Impact Scale, Patient Determined Disease Steps (PDDS); SC: subcutaneous; SD, standard deviation.

**Table 2:** Reasons for study discontinuation.

Reason for Discontinuation	Baseline to 12-Month Visit	Between 12- and 24-Month Visits
Adverse event-related	7	2
Participant’s decision	6	8
Investigator’s decision	3	9
Lost to follow up	3	1
Other	2	4
Could not take tests, could not be examined Disease progression to secondary progressive MS MRI progression, lack of efficacy MS relapse, lack of efficacy		
No answer	17	4
Total	38	28

MRI, magnetic resonance imaging.

**Table 3:** BICAMS outcomes.

Parameter	Baseline			Month 12			Month 24			Repeated Measures ANOVA
	Mean	SD	*n*	Mean	SD	*n*	Mean	SD	*n*	*p*
SDMT	48.46	12.17	129	49.39	13.59	129	51.88	13.35	129	<0.001
BVMT	27.92	6.48	122	29.29	7.07	122	29.91	6.14	122	0.004
CVLT	58.48	10.08	116	60.93	11.87	116	63.84	11.05	116	<0.001

SDMT: Symbol Digit Modalities Test; BVMT: Brief Visuospatial Memory Test; CVLT: California Verbal Learning Test II (CVLT-II).

**Table 4:** Employment status of Teri-REAL patients by visit.

Current Employment Status		Employed Full-Time	Employed Part-Time	Not Currently Employed	Total
Baseline	Count	124	24	58	206
	%	60%	12%	28%	100%
12-month visit	Count	103	23	51	177
	%	58%	13%	29%	100%
24-month visit	Count	84	20	29	133
	%	63%	15%	22%	100%

**Table 5:** Test battery used in Teri-REAL.

Parameter	Test	Description	Reference
Quality of Life	MSQoL-54	MS-specific test with 54 questions related to physical health and mental health. Scores for each scale range from 0 to 100, with a higher score indicating improved quality of life.	[32,33]
Relapse	ARR	Calculated from number of MS relapses over the 2 years of the study	
Disability progression	PDDS	Scale focusing on how patients walk, with a higher score denoting greater disability	[24,25]
Fatigue	FIS	Impact of fatigue on cognitive, physical, and psychosocial function, score range 0 to 160, with a higher score indicating more severe fatigue levels. Adapted for Hungarian native speakers by Losonczi et al., 2011	[34,35,36]
Depression	BDI	21 questions, score range 0 to 63, with a higher score indicating a greater level of depression	[37,38]
Cognition	BICAMS	Incorporating tests for mental processing speed (Symbol Digit Modalities Test (SDMT)), visual memory (Brief Visuospatial Memory Test (BVMT)) and verbal learning (California verbal learning test II (CVLT-II). Adapted for use with Hungarian native speakers by Sandi et al., 2015	[29,39]
Health economic outcomes	HRPQ	Changes in employment status (full-time, part-time, not employed) and absenteeism (the number of days participants missed work, school, or regular daily activities)	[40]

ARR: annualised relapse rate; BDI: Beck Depression Inventory; BICAMS: Brief International Cognitive Assessment for Multiple Sclerosis; BVMT: Brief Visuospatial Memory Test; CVLT: California verbal learning test; FIS: Fatigue Impact Scale; MSQoL-54: Multiple Sclerosis Quality of Life-54; PDDS: Patient Determined Disease Steps; SDMT: Symbol Digit Modalities Test.

## Data Availability

Data is contained within the article.

## References

[1] Multiple Sclerosis International Federation (2020). Atlas of MS.

[2] Giovannoni G., Butzkueven H., Dhib-Jalbut S., Hobart J., Kobelt G., Pepper G., Sormani M.P., Thalheim C., Traboulsee A., Vollmer T. (2016). Brain health: Time matters in multiple sclerosis. Mult. Scler. Relat. Disord..

[3] Benedict R.H., Wahlig E., Bakshi R., Fishman I., Munschauer F., Zivadinov R., Weinstock-Guttman B. (2005). Predicting quality of life in multiple sclerosis: Accounting for physical disability, fatigue, cognition, mood disorder, personality, and behavior change. J. Neurol. Sci..

[4] Mitchell A.J., Benito-León J., González J.M.M., Rivera-Navarro J. (2005). Quality of life and its assessment in multiple sclerosis: Integrating physical and psychological components of wellbeing. Lancet Neurol..

[5] Hoogs M., Kaur S., Smerbeck A., Weinstock-Guttman B., Benedict R.H.B. (2011). Cognition and Physical Disability in Predicting Health-Related Quality of Life in Multiple Sclerosis. Int. J. MS Care.

[6] Kratz A.L., Braley T.J., Foxen-Craft E., Scott E., Murphy J.F., Murphy S.L. (2017). How Do Pain, Fatigue, Depressive, and Cognitive Symptoms Relate to Well-Being and Social and Physical Functioning in the Daily Lives of Individuals with Multiple Sclerosis?. Arch. Phys. Med. Rehabil..

[7] Miller A.E. (2017). Teriflunomide in multiple sclerosis: An update. Neurodegener Dis. Manag..

[8] Miller A.E. (2021). An updated review of teriflunomide’s use in multiple sclerosis. Neurodegener Dis. Manag..

[9] Sanofi-Aventis (2013). AUBAGIO (Teriflunomide) Summary of Product Characteristics [Internet]. https://www.ema.europa.eu/en/documents/product-information/aubagio-epar-product-information_en.pdf.

[10] O’Connor P.W., Li D., Freedman M.S., Bar-Or A., Rice G.P.A., Confavreux C., Paty D.W., Stewart J.A., Scheyer R., Teriflunomide Multiple Sclerosis Trial Group (2006). A phase II study of the safety and efficacy of teriflunomide in multiple sclerosis with relapses. Neurology.

[11] O’Connor P., Wolinsky J.S., Confavreux C., Comi G., Kappos L., Olsson T.P., Benzerdjeb H., Truffinet P., Wang L., Miller A. (2011). Randomized Trial of Oral Teriflunomide for Relapsing Multiple Sclerosis. N. Engl. J. Med..

[12] Confavreux C., O’Connor P., Comi G., Freedman M.S., Miller A.E., Olsson T.P., Wolinsky J.S., Bagulho T., Delhay J.L., Dukovic D. (2014). Oral teriflunomide for patients with relapsing multiple sclerosis (TOWER): A randomised, double-blind, placebo-controlled, phase 3 trial. Lancet Neurol..

[13] Vermersch P., Czlonkowska A., Grimaldi L.M., Confavreux C., Comi G., Kappos L., Olsson T.P., Benamor M., Bauer D., Truffinet P. (2014). Teriflunomide versus subcutaneous interferon beta-1a in patients with relapsing multiple sclerosis: A randomised, controlled phase 3 trial. Mult. Scler J..

[14] Miller A.E., Macdonell R., Comi G., Freedman M.S., Kappos L., Mäurer M., Olsson T.P., Wolinsky J.S., Bozzi S., Dive-Pouletty C. (2014). Teriflunomide reduces relapses with sequelae and relapses leading to hospitalizations: Results from the TOWER study. J. Neurol..

[15] Coyle P.K., Khatri B., Edwards K.R., Meca-Lallana J.E., Cavalier S., Rufi P., Benamor M., Brette S., Robinson M., Gold R. (2017). Patient-reported outcomes in relapsing forms of MS: Real-world, global treatment experience with teriflunomide from the Teri-PRO study. Mult. Scler. Relat. Disord..

[16] Hestvik A.L., Frederiksen J., Nielsen H.H., Torkildsen Ø., Eek C., Huang-Link Y., Haghighi S., Poole E.M., Tsai J.A., Kant M. Teri-LIFE: An Observational Study of Quality of Life in Patients with Relapsing Remitting Multiple Sclerosis Treated with Teriflunomide in the Nordic Region. Proceedings of the 35th Congress of the European Committee for Treatment and Research in Multiple Sclerosis.

[17] Kallmann B.A., Tiel-Wilck K., Kullmann J.S., Engelmann U., Chan A. (2019). Real-life outcomes of teriflunomide treatment in patients with relapsing multiple sclerosis: TAURUS-MS observational study. Ther. Adv. Neurol. Disord..

[18] Bucello S., Annovazzi P., Ragonese P., Altieri M., Barcella V., Bergamaschi R., Bianchi A., Borriello G., Buscarinu M.C., Callari G. (2021). Real world experience with teriflunomide in multiple sclerosis: The TER-Italy study. J. Neurol..

[19] Dardiotis E., Perpati G., Nikolaidis I., Tzanetakos D., Deretzi G., Kilidireas C., Mitsikostas D.D., Hadjigeorgiou G., Grigoriadis N. Real-world assessment of quality of life through patient-reported outcomes in relapsing-remitting multiple sclerosis patients treated with teriflunomide for two years. Outcomes of the AURELIO study in Greece. Proceedings of the 37th Congress of the European Committee for Treatment and Research in Multiple Sclerosis (ECTRIMS).

[20] Dardiotis E., Perpati G., Borsos M., Nikolaidis I., Tzanetakos D., Koutlas E., Kilidireas C., Mitsikostas D.D., Hadjigeorgiou G., Grigoriadis N. Teriflunomide improves quality of life in a Greek cohort of relapsing-remitting multiple sclerosis patients switched from injectables: Subgroup analysis of previously-treated patients in the AURELIO study. Proceedings of the 37th Congress of the European Committee for Treatment and Research in Multiple Sclerosis (ECTRIMS).

[21] De Sèze J., Devy R., Planque E., Delabrousse-Mayoux J.P., Vandhuick O., Kabir M., Gherib A. (2021). Fatigue in teriflunomide-treated patients with relapsing remitting multiple sclerosis in the real-world Teri-FAST study. Mult. Scler. Relat. Disord..

[22] Alroughani R., Inshasi J., Al Khawajah M., Ahmed S.F., Al Malik Y., Alkhabouri J., Shatila A., Aljarallah S., Cupler E.J., Qureshi S.A. (2022). Real-world effectiveness and safety profile of teriflunomide in the management of multiple sclerosis in the Gulf Cooperation Council countries: An expert consensus narrative review. Mult. Scler. J. Exp. Transl. Clin..

[23] Biernacki T., Sandi D., Kincses Z.T., Füvesi J., Rózsa C., Mátyás K., Vécsei L., Bencsik K. (2019). Contributing factors to health-related quality of life in multiple sclerosis. Brain Behav..

[24] Hohol M.J., Orav E.J., Weiner H.L. (1999). Disease steps in multiple sclerosis: A longitudinal study comparing disease steps and EDSS to evaluate disease progression. Mult. Scler..

[25] Hohol M.J., Orav E.J., Weiner H.L. (1995). Disease steps in multiple sclerosis: A simple approach to evaluate disease progression. Neurology.

[26] Patti F., Amato M.P., Trojano M., Bastianello S., Tola M.R., Picconi O., Cilia S., Cottone S., Centonze D., Gasperini C. (2011). Quality of life, depression and fatigue in mildly disabled patients with relapsing-remitting multiple sclerosis receiving subcutaneous interferon beta-1a: 3-year results from the COGIMUS (COGnitive Impairment in MUltiple Sclerosis) study. Mult. Scler. J..

[27] Rendas-Baum R., Yang M., Cattelin F., Wallenstein G.V., Fisk J.D. (2010). A novel approach to estimate the minimally important difference for the fatigue impact scale in multiple sclerosis patients. Qual. Life Res..

[28] Button K.S., Kounali D., Thomas L., Wiles N.J., Peters T.J., Welton N.J., Ades A.E., Lewis G. (2015). Minimal clinically important difference on the Beck Depression Inventory-II according to the patient’s perspective. Psychol. Med..

[29] Sandi D., Rudisch T., Füvesi J., Fricska-Nagy Z., Huszka H., Biernacki T., Langdon D.W., Langane É., Vécsei L., Bencsik K. (2015). The Hungarian validation of the brief international cognitive assessment for multiple sclerosis (BICAMS) battery and the correlation of cognitive impairment with fatigue and quality of life. Mult. Scler. Relat. Disord..

[30] Filser M., Schreiber H., Pöttgen J., Ullrich S., Lang M., Penner I.K. (2018). The Brief International Cognitive Assessment in Multiple Sclerosis (BICAMS): Results from the German validation study. J. Neurol..

[31] Strober L.B., Chiaravalloti N., DeLuca J. (2018). Should I stay or should I go? A prospective investigation examining individual factors impacting employment status among individuals with multiple sclerosis (MS). Work.

[32] Vickrey B.G., Hays R.D., Harooni R., Myers L.W., Ellison G.W. (1995). A health-related quality of life measure for multiple sclerosis. Qual. Life Res..

[33] Füvesi J., Bencsik K., Benedek K., Mátyás K., Mészáros E., Rajda C., Losonczi E., Fricska-Nagy Z., Vécsei L. (2008). Cross-cultural adaptation and validation of the “Multiple Sclerosis Quality of Life Instrument” in Hungarian. Mult. Scler..

[34] Fisk J.D., Ritvo P.G., Ross L., Haase D.A., Marrie T.J., Schlech W.F. (1994). Measuring the functional impact of fatigue: Initial validation of the fatigue impact scale. Clin. Infect Dis..

[35] Losonczi E., Bencsik K., Rajda C., Lencsés G., Török M., Vécsei L. (2011). Validation of the Fatigue Impact Scale in Hungarian patients with multiple sclerosis. Qual. Life Res..

[36] Hobart J., Cano S., Baron R., Thompson A., Schwid S., Zajicek J., Andrich D. (2013). Achieving valid patient-reported outcomes measurement: A lesson from fatigue in multiple sclerosis. Mult. Scler..

[37] Beck A.T., Steer R.A., Brown G.K. (1996). Manual for the Beck Depression Inventory-II.

[38] Beck A.T., Ward C.H., Mendelson M., Mock J., Erbaugh J. (1961). An inventory for measuring depression. Arch. Gen. Psychiatry.

[39] Langdon D.W., Amato M.P., Boringa J., Brochet B., Foley F., Fredrikson S., Hämäläinen P., Hartung H.P., Krupp L., Penner I.K. (2012). Recommendations for a brief international cognitive assessment for multiple sclerosis (BICAMS). Mult. Scler. J..

[40] Kumar R.N., Hass S.L., Li J.Z., Nickens D.J., Daenzer C.L., Wathen L.K. (2003). Validation of the Health-Related Productivity Questionnaire Diary (HRPQ-D) on a sample of patients with infectious mononucleosis: Results from a phase 1 multicenter clinical trial. J. Occup. Environ. Med..

[41] Osterberg L., Blaschke T. (2005). Drug therapy—Adherence to medication. N. Engl. J. Med..

